# Cube - An Online Tool for Comparison and Contrasting of Protein Sequences

**DOI:** 10.1371/journal.pone.0079480

**Published:** 2013-11-20

**Authors:** Zong Hong Zhang, Aik Aun Khoo, Ivana Mihalek

**Affiliations:** Bioinformatics Institute, Agency for Science, Technology and Research, Singapore; Indian Institute of Science, India

## Abstract

When comparing sequences of similar proteins, two kinds of questions can be asked, and the related two kinds of inference made. First, one may ask to what degree they are similar, and then, how they differ. In the first case one may tentatively conclude that the conserved elements common to all sequences are of central and common importance to the protein's function. In the latter case the regions of specialization may be discriminative of the function or binding partners across subfamilies of related proteins. Experimental efforts - mutagenesis or pharmacological intervention - can then be pointed in either direction, depending on the context of the study.

Cube simplifies this process for users that already have their favorite sets of sequences, and helps them collate the information by visualization of the conservation and specialization scores on the sequence and on the structure, and by spreadsheet tabulation. All information can be visualized on the spot, or downloaded for reference and later inspection.

Server homepage: http://eopsf.org/cube

## Introduction

Bioinformaticians have by now enjoyed almost two decades of publicly available protein comparison software and servers. In Cube, we shift somewhat the emphasis, and in addition to presenting our work in a way accessible to a bioinformatician, we address the needs of researchers who have no particular bioinformatics inclination, and for whom the sequence comparison is one of many steps in designing a biochemical or molecular biology experiment. In particular, Cube is structured to highlight the notion that conservation and specialization are two complementary pieces of information. Cube offers them for inspection side-by-side.

To place Cube on the map of the field, we first look at the biology involved, then discuss briefly how bioinformaticians detect and describe it, and how they disseminate their work.

### Evolutionary behavior of biological sequences and the practical value of its analysis

Comparative analysis of DNA or protein sequences relies on an intuitively appealing mechanistic model of their evolution. It starts as a random process in which every region has an equal *a priori* chance of mutating. However, mutations that negatively impact a functionally important region get cleared out of the population.

Evolution will thus reduce the number of residue types observable at each position to the set which is allowable by the function. A thorough and illuminating analysis of the evolutionary process at work on the molecular level can be found in the body of work lead by J.H. Miller [1], [2]. Nowadays, we can reproduce and trace the process in the lab [3]. Conversely, when we analyze conservation of residues or nucleotides, we are reverse engineering the nature-devised system, and looking for plausible functional explanation for why particular residues are conserved [4].

Furthermore, noting that a prominent mechanism of genome evolution is gene duplication, we may enquire which of the copies (termed paralogues) changes to acquire new function [5]. We can look for residues that distinguish therwise similar groups of genes or proteins. These may, but do not need to be conserved in both paralogous groups [6]. After the gene duplication, the rate of evolution may stay the same in the two newly-founded branches (homotachy, in the fanciful terminology of [7], or type II divergence [6]), but is in general free to proceed at different rates (heterotachy, type I divergence). As a limiting case of the former, a position may be conserved as a different residue type in each of the branches (constant-but-different [8], discriminant [9]), or even, as a further extreme, conserved across two groups of related proteins. In any case, locating positions with markedly different evolutionary behavior in different paralogues can be used to understand and inform redesign of protein function [10].

There are several practical problems to solve, though, to get meaningful results out of sequence comparison. Focusing on the word “conserved” one might note that it carries a hidden catch: it makes sense only when coupled with the definition of the set of sequences to which it applies. (Conserved in all protein kinases or conserved in CK1 group? Conserved in all vertebrates, or in mammals only?) The problem is twofold: we have to decide what defines the class of sequences within which we want to look for the conservation, and, then, we need to find those and only those sequences that belong to the class that we want to study.

While the patterns of conservation or specialization are not hard to appreciate once they are pointed out, they might be difficult to analyze systematically by a human observer - the alignment of one hundred vertebrate genes can easily approach a megabyte of data. Therefore, we would like to have ways to detect and classify of evolutionary behavior computationally.

### Methods and their implementations, servers and databases of pre-calculated results

When bioinformaticians develop methods for detecting any particular type of evolutionary behavior, the fundamental way in which they present their work is by publishing the method - the scoring function or the algorithm. This is a compact way, usually involving some algebra, for explaining what the method does. At this point the methods may remain nameless. The names get attached later in the process - to the implementations, and even more often, to the servers. Implementation - the realization of the algorithm as a program is sometimes offered for download. If well written, this is the ultimate documentation for a method.

However, using an implementation directly is a task for aficionados. Servers provide shortcuts for a broader audience - they hide the implementational details from the user, and sometimes combine several sources of information. They differ widely in the way they present the output - from plain text tables that appear in the browser, to automatically generated printable reports and embedded visualization tools. It is notable however that the value expected to be added by the server increases as the field matures.

Sometimes the involved pipeline is so complicated, prone to breaking down, difficult to completely automate, or just time-consuming to complete, that the authors decide to present their results in the form of a database of pre-calculated results. The drawback of a database is that its content is fixed, and it does not allow the interested user to inquire how a change in the input data affects the offered conclusions.

Table 1 compiles (in an admittedly non-exhaustive way) method/server/database references for several notable takes on the protein sequence comparison. It also places Cube in its broader context.

**Table 1:** Comparison of several applications for comparison of protein sequences.

Name	Evolutionary behavior	Algorithm or method	Database	Server
Valdar	(degree of) variability	[11], [12]		ScoreCons^1^
rate4site, ConSurf	variability	[13]	[14]	[15], [16]
AMAS, integer- and real-valued ET	variability	[17]–[19]	[20]	[21], [22]
INTREPID	variability; type II div	[23]		[24]
FunShift	type I div	[25]	[26]	
Diverge	type I and type II div	[6], [27], [28]		
SDP	type II div	[29]		[30]
Treedet	type II div	[31]		[32]
SPEER	type II div	[13], [33]		[34]
Multi-RELIEF	type II div	[35]		[35]
Capra & Singh	type II div	[36]		
Cube	variability; type I and II div	[9], [19]	[37]	this work

The table compiles the name under which a method is most often referred to, the type of evolutionary method it captures, and the references for the original (method) publication, as well as for the accompanying database and/or server publications, where applicable. “Variability” stands for the “degree of variability.” The table is not an exhaustive overview of the field, but, rather, illustrates the following. (i) Bioinformatics applications are usually presented as an algorithm and its application (third column), sometimes as a database of pre-calculated results, and sometimes as a server. Cube, described in this work, is a server. (ii) Furthermore, as of this writing, Cube is unique in that it provides a heuristic scoring both for the overall degree of variability, and for the type I and type II divergence. (iii) Type I divergence does seem to have the thinnest coverage in the literature, and is tackled by Cube.

1 http://www.ebi.ac.uk/thornton-srv/databases/cgi-bin/valdar/scorecons_server.pl.

### Why Cube

It should be noted in the light of the above discussion that Cube is neither a method, nor a database. It is a server, using several methods to calculate on the spot conservation and specialization scores for the provided input. The drawback of this fact is that the users need to provide their own set of sequences for the analysis, which shifts part of the work on the users themselves. At the same time, this offers a possible advantage, because the users can provide the input from any kingdom of life, and group it according to any rule that may as well be unknown to the server. For the users working on vertebrate proteins, it might be of interest that Cube has a sister database of pre-calculated results, Cube-DB [37], with the comparison limited to vertebrate sequences available in ENSEMBL [38].

Behind the server are two pieces of code (available from the server's homepage) implementing several conservation detection methods [12], [19], [39] and one specialization detection method [9]. The specialization method implemented in Cube allows description of both divergence type I and type II events. Cube is a lightweight application with the aim of presenting our work in several formats that we have found to be practical in development and planning of experiments (mutagenesis experiments in particular): tabulation, mapping on the structure, and the sequence (by creating an image that can further be annotated). It leaves the user fully in control over the sequences that the analysis is based on. It is currently unique in that it places side-by-side and invites the contemplation of three types of evolutionary behavior: conservation and type I and type II specialization, conserved vs. determinant and discriminating residues.

We devote the following sections to more detailed description of methods and presentation of results in Cube.

## Methods

Cube provides an interface to two scoring programs, one focusing on the conservation within a set of sequences, and the other on the specialization across several families. Rather than attempting to compound all the data - such as mutational propensity, spatial location, and biochemical properties of a residue - in a single score, we present them side by side, and let the user decide on their synergistic importance.

The scores implemented in Cube are all heuristics (to be distinguished from the algorithms that probabilistically model the underlying evolutionary process [6], [13]). They assign a single score to each position in the alignment, and assume the positions to be independent. They are “frequentist,” in that the inference is based on distribution of frequencies 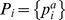 with which the amino acid type 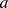 appears in the alignment column 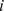. In Cube, all scores are turned into ranks, which are in turn expressed as the top fraction they represent.

### Conservation scoring

The user can choose between several heuristic, time-proven methods: real-valued ET [19], and integer-valued ET [39], majority fraction [40], Shannon's information entropy, and Valdar's score, the last three described in [12]. All of these scores have the same common structure, where to the alignment position 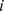 a value 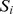 is assigned, such that 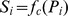. That is, the value of the score is a function of the frequency distribution of the amino acid types seen at this position. For example the majority fraction takes 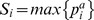, the largest fraction seen at the position 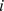, and Shannon entropy takes 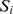 to be 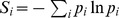.

Biochemical similarity of residues can be taken into account by using a reduced alphabet of amino acids, or by using BLOSUM [41] similarity in the case of Valdar's method. In these cases the function 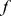 is parametrized in a way that depends on type similarity. This parametrization is independent of the position 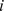. Valdar's score is also the only one that attempts to correct for the uneven taxonomical sampling in the provided sequence set. rvET and ivET scores take the underlying similarity tree structure into account.

### Specialization scoring

The specialization scoring is provided in two flavors. In the simpler approach, with the score termed “cube” and described in [9], the positions are highlighted for which the overlap in distribution of amino acid types differs between the provided groups. This score is unaware of the possible relevance of biochemical similarity of some residues types. Alternatively, thus, the score that corrects for the effect is provided (“cube with similarity”). As in the case of conservation, the scoring function can be written as 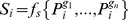, the difference being that 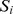 is now the function of 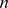 distributions in 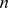 protein groups, 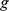. The similarity is incorporated in the score by comparing the overlap with the expected overlap for (hypothetical) freely evolving residue distributions in the two groups. The scoring function does not use BLOSUM directly, but derives an evolutionary law for the distribution 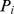, such that after very long hypothetical time, every initial distribution 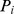 converges to an equilibrium distribution which reproduces BLOSUM [42]. The overlap in residue type distribution between all group pairs is turned into two related but different pieces of information - discriminant and determinant score. The former rewards positions that are unique in one of the groups, while the latter seeks rarer cases in which a position is unique for each of the groups.

### The scope and the limitations

The purpose of the methods implemented in Cube is to highlight residues exhibiting certain evolutionary behavior. The scores it uses are qualitative, and their absolute values carry no intrinsic meaning. Furthermore, the relative ranking of residues depends much more strongly on taxonomical sampling and the quality of the alignment, then on the precise choice of the method. In addition, when scoring the alignment positions the question of homology/orthology/paralogy arises. Faulty classification, again, may have more impact on the output than the method choice.

### Implementation

The server is a mid-sized processing pipeline implemented in Perl/CGI/JavaScript, and was tested on all of the most popular web browsers. The scoring methods are implemented in C, and the code is available on the server's webpage.

### Dependencies

Cube server uses MUSCLE [43] and MAFFT [44] to align sequences, and DSSP [45] to estimate the surface accessibility of individual residues. It also produces visualization for download, as a PyMOL session. [DeLano, W. (2002). The PyMOL Molecular Graphics System. (http://www.pymol.org). See also http://www.pymolwiki.org/index.php/Practical_Pymol_for_Beginners#Sessions.]

## Results and Discussion

### User's perspective

In designing Cube, we tried deliberately to keep it's interface lean. It has two main entry points. Starting from the dashboard page, the user can choose to do conservation or specialization analysis.

### Conservation module

The only required input is a set of sequences in fasta format. Optionally, the sequences can be pre-aligned (the server accepts fasta and msf formats), and the reference sequence specified. In addition, the structure can be provided, and the default scoring method changed.

**Figure 1:**
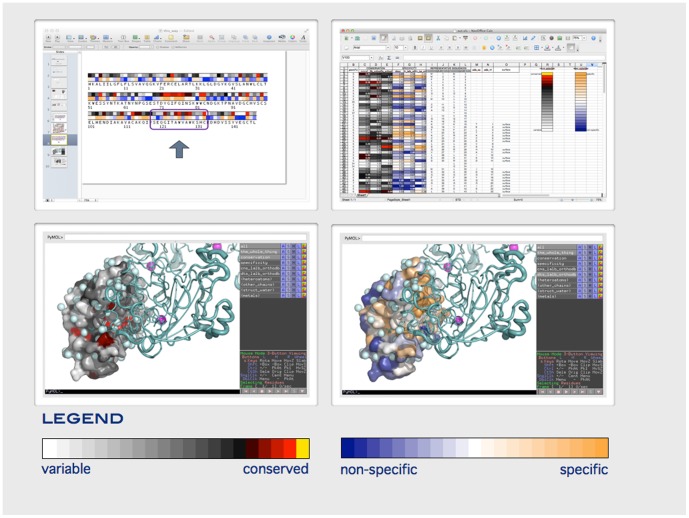
Visualization in Cube. Clockwise from top left: one dimensional map in png format, spreadsheet tabulation of conservation, specialization and annotation provided by the user, specialization mapped on the structure, and conservation mapped on the structure. The example shown: specialization between lysozyme C and 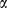-lactalbumin. (See http://eopsf.org/cube/help/worked_examples/spec_examples.html.).

The server produces a 1D conservation map (the conservation score color coded and mapped on the sequence) in the png format, the tabulated information (in xls format), and the conservation mapped onto the structure (as a PyMol session, see the ‘Dependencies’ subsection in ‘Methods,’ above), Fig. 1. A consistent color coding is used in all three forms of the output. The users are invited to provide any information that they already may have about the protein residues (such as transmembrane regions, post-translational modifications sites, catalytic residues and similar), numbered according to any sequence in the alignment. This information is added to the downloadable table, alongside the conservation score, residue type, and surface accessibility information.

When the structure (in PDB [46] format) is provided, the conservation score is mapped onto either the first chain or the user-specified chain in the provided PDB file. The server generates a PyMol session file in which the remaining peptide chains and ligands are indicated using a cartoon representation. From within the session, the poorly scoring residues can be hidden to emphasize the clusters of the most conserved residues.

### Specialization module

The user is required to upload sequences already divided into meaningful groups. The groups can be arbitrary, but typically they are expected to represent paralogous families of proteins in comparable taxonomical samples, or protein orthologues divided into clearly distinct taxonomical groups.

In the output (Fig. 1), the specialization scores are shown side-by-side with the conservation values (Shannon entropy) for each residue, both in the tabulated output (xls spreadsheet) as well as mapped on the structure (Pymol session). In the spreadsheet the results are laid out literally side-by-side in the adjacent columns. In the Pymol session, the menu on the right allows switching between the two views. The scores are also immediately shown in the browser, and available as a downloadable 1D map in the png format, and as an html version of the output table.

### Documentation

The server comes with extensive help pages, worked examples, and on-the-spot help in the form of “mouseover” events, provided in the hope that it will find its place in biochemists', and molecular biologists' toolbox.
